# Italian validation of the ACSS-FAD in a sample of university students

**DOI:** 10.1192/j.eurpsy.2021.1579

**Published:** 2021-08-13

**Authors:** F. Dolenz, S. Magliocca, A. Silva, F. Madeddu, R. Calati

**Affiliations:** 1 Psychology, University of Milano-Bicocca, Rapallo (GE), Italy; 2 Psychology, University of Milano-Bicocca, Barlassina, Italy; 3 Psychology, University of Milano-Bicocca, Romanengo, Italy; 4 Psychology, University of Milano-Bicocca, Milan, Italy; 5 Psychiatry, Nîmes University Hospital, Nîmes, France

## Abstract

**Introduction:**

Suicide is one of the most relevant cause of death especially in young populations. The Interpersonal Theory of Suicide (Joiner, 2005) is an important contribution tends to explain variability in suicidal behavior, particularly the difference between suicidal ideation and suicide attempt.

**Objectives:**

This study aimed at the Italian validation of the Acquired Capability for Suicide Scale – Fearlessness About Death (ACSS-FAD), assessing fearlessness about death, one of the facets of the acquired capability (AC) to commit suicide as postulated by the Interpersonal Theory of Suicide by Thomas Joiner.

**Methods:**

This cross-sectional research was conducted on a sample of university students (n = 458) assessed with a battery including ACSS-FAD. The sample was evaluated for the presence of suicidal ideation and suicide risk. Factor structure, internal consistency and convergent/divergent validity of the scale were assessed.

**Results:**

One-factor structure with good internal consistency (Cronbach’s α: 0.84) was derived. ACSS-FAD correlated with suicidal ideation and suicidal risk, and there was a tendency towards significance considering its capacity to discriminate between those who had a history of suicide attempts and those who did not. 4.1% of the sample attempted suicide at least one time. The tool showed good convergent/discriminant validity results, but the relationship between ACSS-FAD and pain needs further investigations.

**Conclusions:**

ACSS-FAD seems to be a useful and valid measure of fearlessness about death especially in young adults, which could be really important to enhance suicide risk assessment.

**Conflict of interest:**

No significant relationships.

